# Components in SLPE Alleviate AD Model Nematodes by Up-Regulating Gene *gst-5*

**DOI:** 10.3390/ijms251810188

**Published:** 2024-09-23

**Authors:** Peng Zhao, Zifu Wang, Shimei Liao, Yangxin Liao, Shijun Hu, Jianchun Qin, Donghua Zhang, Xiaohui Yan

**Affiliations:** 1 Key Laboratory of Forest Disaster Warning and Control in Yunnan Province, Southwest Forestry University, Kunming 650224, China; aligaduo103849724@163.com (P.Z.); wang_zi_fu@163.com (Z.W.); liao5253leg@163.com (S.L.); lyx258xxxxx@163.com (Y.L.); 2 Key Laboratory of Biodiversity Conservationin Southwest China (State Forestry Administration), Southwest Forestry University, Kunming 650224, China; shijunhu@swfu.edu.cn; 3 College of Plant Science, Jilin University, Xi’an Road No. 5333, Changchun 130062, China; qinjc@jlu.edu.cn

**Keywords:** *Salvia leucantha*, Alzheimer’s disease, *Caenorhabditis elegans*, transcriptome, RNAi

## Abstract

*Salvia leucantha* is a perennial herb of the genus *Salvia* in the family Labiatae, which has a wide range of biological activities, mainly including inhibition of acetylcholinesterase, antibacterial, and anti-inflammatory activity. To explore the protective effects and mechanism of action of *S. leucantha* on Alzheimer’s disease (AD), the anti-AD activity of SLE (extracts of *S. leucantha*) was determined by using a transgenic *Caenorhabditis elegans* (*C. elegans*) model (CL4176). Analyses included paralysis assay, phenotypic experiments, transcriptome sequencing, RNA interference (RNAi), heat shock assays, and gas chromatography-mass spectrometry (GC-MS). SLPE (*S. leucantha* petroleum ether extract) could significantly delay CL4176 paralysis and extend the longevity of *C. elegans* N2 without harmful effects. A total of 927 genes were significantly changed by SLPE treatment in *C. elegans,* mainly involving longevity regulatory pathways—nematodes, drug metabolism—cytochrome P450, and glutathione metabolic pathways. RNAi showed that SLPE exerted its anti-AD activity through up-regulation of the gene *gst-5*; the most abundant compound in SLPE analyzed by GC-MS was 2,4-Di-tert-butylphenol (2,4-DTBP), and the compound delayed nematode paralysis. The present study suggests that active components in *S. leucantha* may serve as new-type anti-AD candidates and provide some insights into their biological functions.

## 1. Introduction

Alzheimer’s disease (AD) is the most prevalent chronic neurodegenerative disorder, affecting a significant population of 5.7 million individuals in the United States alone. This number is projected to escalate to 13.8 million by 2050 [1]. The disease is characterized by two key pathological features, namely extracellular amyloid plaques and intracellular neurofibrillary tangles [2]. The etiology of AD is multifaceted, and the therapeutic approaches vary. Some examples include cholinesterase inhibitors like donepezil [3], lisdexamfetamine [4], and galantamine [5], which target the cholinergic deficit hypothesis. Additionally, memantine [6], an N-methyl-D-aspartate receptor blocker, and the antioxidant drug silymarin [7], which has been investigated about the oxidative stress doctrine, constitute other pharmacological treatments. However, none of these interventions can completely prevent or cure the disease; they can only mitigate its progression, and some may pose potential side effects. Consequently, there is an urgent need to conduct further research on anti-AD drugs.

*Caenorhabditis elegans* (*C. elegans*), commonly found in temperate soil environments, has emerged as a valuable model organism since its first application in developmental biology research during the 1960s [8]. Its versatility extends to various fields, including studies related to obesity, aging, developmental processes, motor functions, and neurodegenerative diseases. In comparison to traditional animal models, *C. elegans* offers several advantages. Notably, it possesses a small body size (approximately 1 mm in length as an adult), a short lifespan (around 21 days in the wild type), and a high reproductive capacity (approximately 300 offspring through autologous fertilization) [9]. Furthermore, the complete genome of *C. elegans* has been fully sequenced and annotated, further enhancing its appeal as a research subject. The genetic accessibility of *C. elegans* is another significant strength, enabling targeted deletions, mutagenesis screens, and genome-scale RNAi screens. As a result, it has become widely employed as an alternative model organism to study genetic mechanisms [10]. The Centre for *C. elegans* Genetics at the University of Minnesota has made a remarkable contribution by providing over 3000 mutant strains of *C. elegans* at an affordable cost. This resource greatly facilitates the utilization of *C. elegans* as a model organism [11].

Plants hold an abundant array of bioactive compounds, making them an invaluable natural resource. Extracting effective medicinal ingredients from plants, such as glycyrrhizin from licorice, polyphenols from cranberry, and diterpenoids from *Salvia divinorum*, has shown promise in alleviating symptoms associated with Alzheimer’s disease, thus emerging as a crucial strategy in the development of anti-AD drugs in recent years [12,13,14]. In addition, studies have demonstrated that compounds derived from *Salvia castanea* possess the ability to significantly delay paralysis symptoms in *C. elegans* [15]. *S. leucantha*, another member of the Salvia genus native to south–central America, including Mexico and other regions, exhibits exceptional heat and drought tolerance, reduced vulnerability to pests and diseases, and boasts a prolonged flowering period, rendering it a trendy choice for garden landscapes [16]. Considering the genus specificity of plant components, we investigated the anti-AD activity of *S. leucantha* and found that the extract of *S. leucantha* can effectively delay the paralytic effect of AD model nematodes.

## 2. Results

### 2.1. SLPE Delays CL4176 Paralysis Symptoms

**Figure 1:**
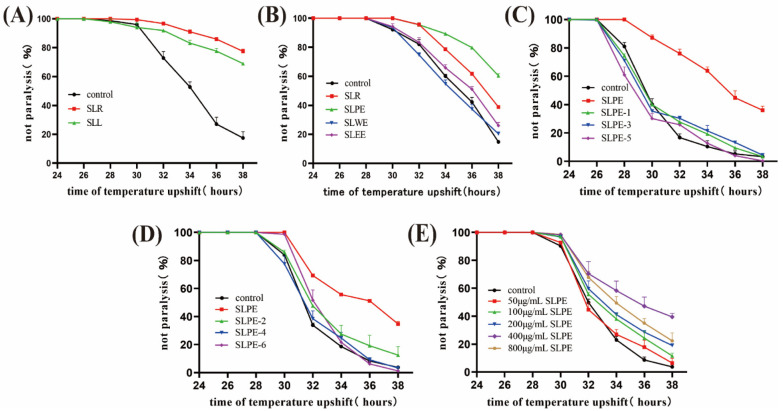
Effects of different extracts from *S. leucantha* on AD model nematodes. SLR: *S. leucantha* ethanol heated reflux extracts. SLL: *S. leucantha* room temperature ultrasonic extracts. SLPE: Petroleum ether extracts of SLR. SLEE: Ethyl acetate extracts of SLR. SLWE: Water extracts of SLR. (**A**) Non-paralysis rates of 100 μg/mL SLR- and SLL-treated CL4176. (**B**) Non-paralysis rates of 100 μg/mL SLR- and SLPE- and SLEE- and SLWE-treated CL4176. (**C**,**D**) Effects of gel column chromatography sample of *S. leucantha* on paralysis of *C. elegans* (100 μg/mL). (**E**) Non-paralysis rates of CL4176 treated with different concentrations of SLPE.

We examined whether *S. leucantha* extract in *C. elegans* CL4176 affects paralysis induced by β-amyloid (Aβ) toxicity. Non-paralysis rates were recorded at 2 h intervals after 24 h of transfer temperature. Figure 1 demonstrates that different extracts of *S*. *leucantha* delayed nematode paralysis in the AD model, compared with the control group, and 400 μg/mL SLPE significantly delayed paralysis, suggesting that SLPE alleviates AD symptoms by counteracting Aβ-induced toxicity. A total of 400 μg/mL SLPE had the best delaying effect and the most stable delaying effect, and this concentration was chosen for all subsequent experiments.

### 2.2. SLPE Extends the Lifespan of C. elegans without Affecting Other Basic Biological Characteristics 

**Figure 2:**
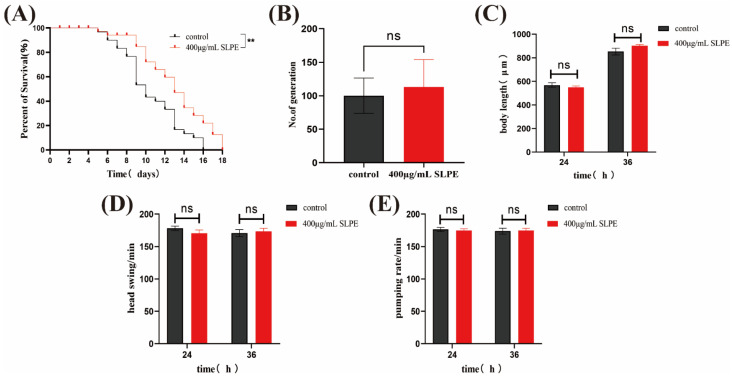
Effects of SLPE on basic biological characteristics of N2 nematodes. (**A**): Lifespan; (**B**): no. of generation; (**C**): body length; (**D**): head swing; (**E**): pumping rate. ** Significantly different from the control worms (*p* < 0.01), “ns” means no significance.

*C. elegans*, with its easily cultivable nature and short life cycle, serves as a robust model for longevity studies [17]. In this research, the wild-type *C. elegans* N2 strain was selected as the subject. The nematodes in the study exhibited free movement and feeding capabilities when alive while showing a rigid, straight state at both ends of the body upon gentle touch with a hair strand when considered deceased. In the control group, the average lifespan of *C. elegans* was 13 days, with a maximum lifespan of 16 days. However, in the experimental group, the average lifespan increased to 15 days, with a maximum lifespan of 18 days. These findings suggest that SLPE can extend the lifespan of *C. elegans*. Notably, N2 nematodes, with an increasing culture time, may experience reduced or compromised reproductive capacity to maintain somatic cell homeostasis [18]. To assess the impact of the drug on *C. elegans*’ reproductive ability and offspring count while reducing Aβ deposition, the number of eggs laid was analyzed. Figure 2 illustrates the results, which revealed no significant difference in the number of eggs produced by the SLPE-treated N2 nematodes, indicating that their reproductive ability remained unaffected. *C. elegans* exhibits various phenotypes directly linked to Alzheimer’s disease (AD), particularly in terms of swallowing speed. As AD progresses, there is a gradual decline in the organism’s overall functioning, resulting in reduced food intake and impaired ability to acquire sufficient nutrients independently [19]. In this study, no significant differences were observed in the growth, development, and survival of the SLPE-treated N2 nematodes, indicating that the treatment does not impact the phenotype or food intake of *C. elegans*.

### 2.3. Effect of SLPE on the Expression of Different Genes and Results of GO and KEGG Enrichment Analysis

A total of 927 differentially expressed genes (|log2(FoldChange)| > 1 and *p* ≤ 0.05) were identified between the treated group (SLPE) and the untreated group (CK), of which 355 were up-regulated and 572 down-regulated in expression. The overall distribution of altered genes was shown in volcano plot (Figure S1) and heatmap of genes (Figure S2) altered by SLPE treatment in *C. elegans*. Differentially expressed genes (DEGs) (*p* < 0.05) were shown in Table S1.

**Figure 3:**
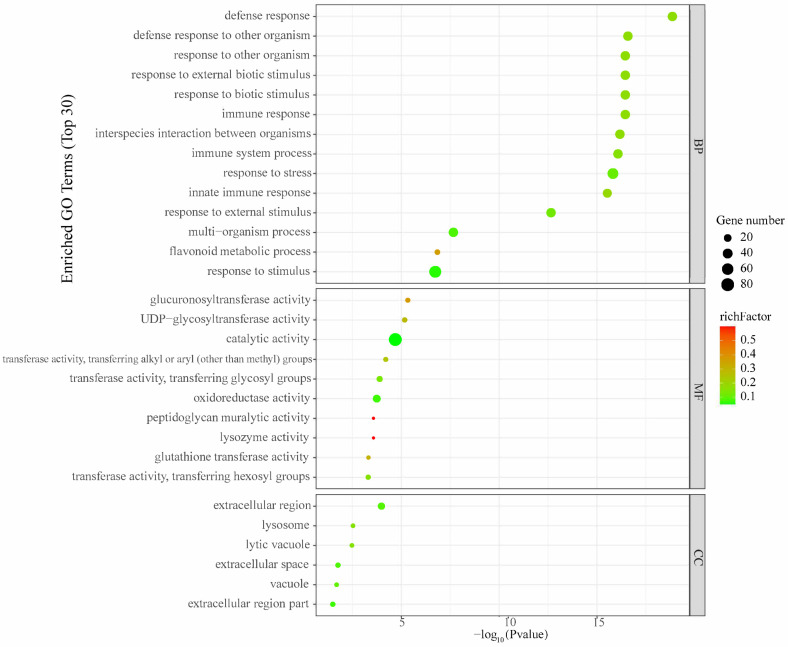
Significant GO terms of DEGs were found by enrichment analyses in *C. elegans* treated with SLPE.

The results of Gene ontology (GO) enrichment that changed due to SLPE treatment are presented in Table S2. As shown in Figure 3, the differential genes obtained were further analyzed by GO enrichment, where the top 30 entries were enriched to contain 14 Biological Process (BP) entries, 6 Cellular Component (CC) entries, and 10 Molecular Function (MF) entries. In the BP analysis, differential genes were mainly enriched in defense response to another organism, response to other organisms, and flavonoid metabolic process; CC analyses revealed that most of the targets of SLPE for the relief of AD nematode paralysis are partially concentrated in the extracellular region and are closely related to the lysosome and lytic vacuole in the cell. Through MF analysis, it can be learned that the targets of SLPE are closely related to the reactions of various enzymes, mainly glucuronosyltransferase activity, UDP–glycosyltransferase activity, oxidoreductase activity, peptidoglycan muralytic activity, amino acid biosynthesis, peptidoglycan muralytic activity, glycosyltransferase activity, oxidoreductase activity, peptidoglycan muralytic activity, biosynthesis of amino acids, glutathione, and other amino acids, glutathione transferase activity, transferase activity, and transferring hexosyl groups. The above results suggest that SLPE acts on multicellular components involved in different cellular response processes, thereby alleviating AD nematode paralysis.

**Figure 4:**
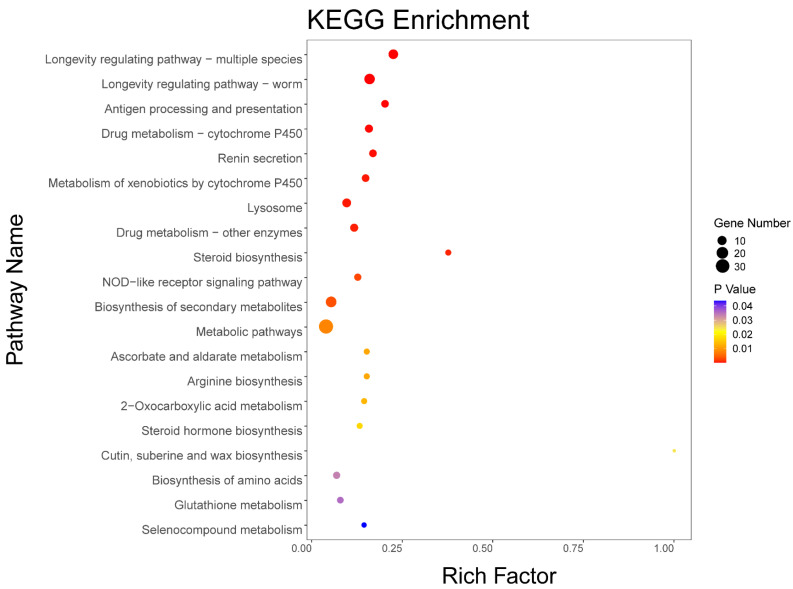
Significant KEGG pathways were identified by enrichment analyses. The rich factor is the proportion of the overall amount of DEG in the various groups to the entire amount of genes discovered in the KEGG analysis.

Kyoto Encyclopedia of Genes and Genomes (KEGG) pathways that were altered by SLPE treatment are shown in Table S3. As shown in Figure 4, The KEGG pathway enrichment analysis yielded 20 pathways that were significantly enriched this time, with differential gene enrichment in the longevity regulating pathway—worm, drug metabolism—cytochrome P450, metabolism of xenobiotics by cytochrome P450, NOD-like receptor signaling pathway, 2-oxocarboxylic acid metabolism, steroid hormone biosynthesis, biosynthesis of amino acids, and glutathione metabolism, suggesting that SLPE is involved in alleviating AD nematode paralysis through the regulation of multiple signal transduction pathways.

### 2.4. PPI Network Analyses of DEGs

**Figure 5:**
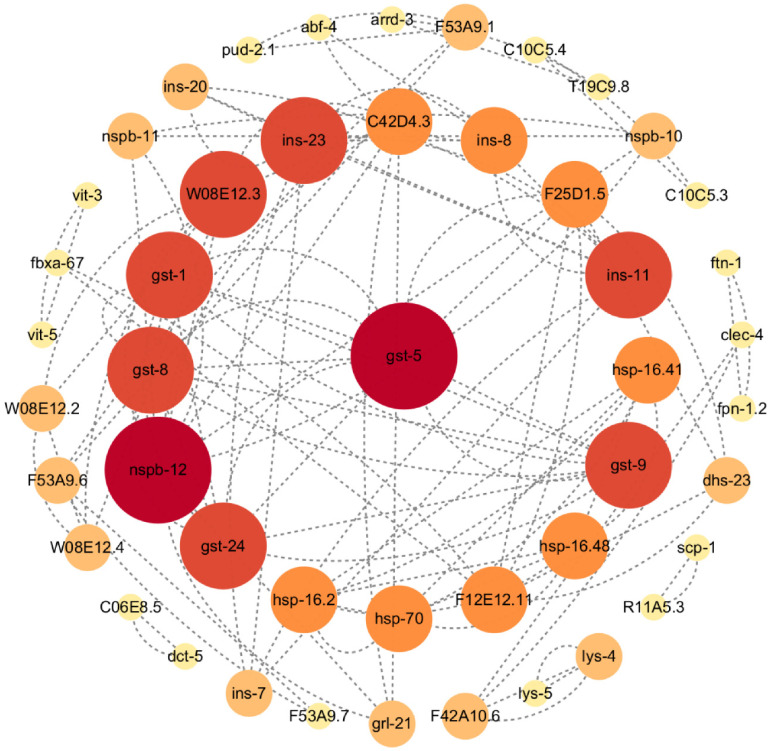
PPI network analyses of DEGs in *C. elegans* treated with SLPE (*p* < 0.05). The magnitude of the genetic degree from greater to lesser is indicated by red shading from darker to lighter.

The protein–protein interaction (PPI) network serves as a valuable tool in identifying genes that encode core proteins with crucial bioregulatory functions [20]. PPI was used to analyze the interrelationships between the differential genes found in SLPE-treated *C. elegans* using the string online database and Cytoscape software. Figure 5 illustrates a total of 54 DEGs (*p* < 0.05, 22 up-regulated genes and 32 down-regulated genes) screened into the PPI network, yielding 47 nodes, and based on the criterion of the degree from the largest to the smallest, it was determined that the gene *gst-5* was the center node gene.

The main protein interaction pathways respond to the *hsp-16.11*, *hsp-16.41*, *hsp-16.48*, and *hsp-70* heat shock protein family, of which *hsp-16.41* and *hsp-16.48* may act as passive ligands to temporarily prevent unfolded protein aggregation, while *hsp-16.11* has been shown to interact with intracellular human β-amyloid protein and prevents its expression, thus exerting an anti-AD effect. The *gst-1*, *gst-5*, *gst-8*, *gst-9,* and *gst-24* glutathione S-transferase family, which is involved in the process of glutathione metabolism, and the human immediate homologs of this group of genes are related to AD; *ins-7*, *ins-11*, *ins-20*, and *ins-23* encode insulin-like peptides of the insulin protein superfamily, which are expressed in neurons and play a role in regulating lifespan, and silencing of *ins-7* by RNAi leads to a significant increase in nematode lifespan; *nspb-10*, *nspb-11*, and *nspb-12* encode group B of the nematode-specific peptide family, of which *nspb-12* affects a variety of genes, including *daf-16* and *daf-2*, and *skn-1*, of which *daf-16* in nematodes is an important transcription factor, a homolog of the human FOXO transcription factor, that regulates nematode resistance [21] and can integrate *igf-1*, tor, ampk, jnk, and other signaling pathways [22]. This triggers transcriptional changes in many genes related to senescence, development, stress, metabolism, and immunity, thereby regulating nematode resistance [23]. The *skn-1* molecular signaling pathway is highly homologous to the mammalian Nrf2 pathway [24]. Conserved phase II detoxification responses can be induced to resist oxidative stress and contribute to nematode longevity [25]. The activation of *skn-1* and *daf-16* as transcription factors induce the expression of a series of anti-stress target genes, thereby increasing the nematode’s resistance to stress. SLPE acts in a variety of ways and exerts its anti-AD activity through the synergistic action of multiple pathways and genes.

### 2.5. SLPE Exerts Anti-AD Effects through Genes Gst-5

Differential up-regulated genes *C44B12.3*, *gst-5*, and *T16G1.6* and down-regulated genes *C10G10.4*, *C10G10.5*, and *F55G11.5* were screened for RNAi experiments to see whether SLPE still had a mitigating effect on paralysis in CL4176.

**Figure 6:**
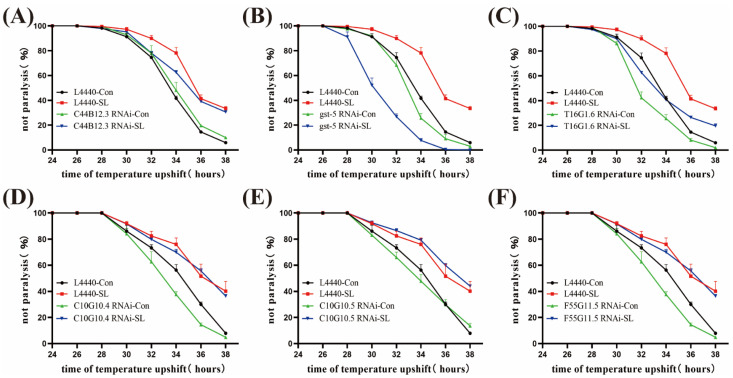
Effects of RNAi on the paralysis of *C. elegans 4176*. (**A**) Effect of SLPE on the paralytic effect of *C44B12.3* RNAi AD nematodes. (**B**) Effect of SLPE on the paralytic effect of *gst-5* RNAi AD nematodes. (**C**) Effect of SLPE on the paralytic effect of *T16G1.6* RNAi AD nematodes. (**D**) Effect of SLPE on the paralytic effect of *C10G10.4* RNAi AD nematodes. (**E**) Effect of SLPE on the paralytic effect of *C10G10.5* RNAi AD nematodes. (**F**) Effect of SLPE on the paralytic effect of *F55G11.5* RNAi AD nematodes.

The delaying effect of SLPE on AD nematode paralysis was significantly reduced only when the expression of genes *gst-5* (*** *p* < 0.001) was knocked down compared to the control (HT115) group. Even at 36 h, there were still surviving nematodes in the control group (Figure 6B). In contrast, all the nematodes in the interfering group were already paralyzed, indicating that SLPE exerts anti-AD activity through genes *gst-5*.

### 2.6. Compound 2,4-DTBP Delays CL4176 Paralysis and Prolongs the Lifespan of C. elegans N2 in the Heat Shock State

NIST-MS spectral libraries were compared, the constituents were analyzed and identified concerning the standard maps, and the area normalization method was used to select those with a matching factor of >85 for constituent content analysis. A total of 10 compounds with a match factor of >85 were identified.

**Figure 7:**
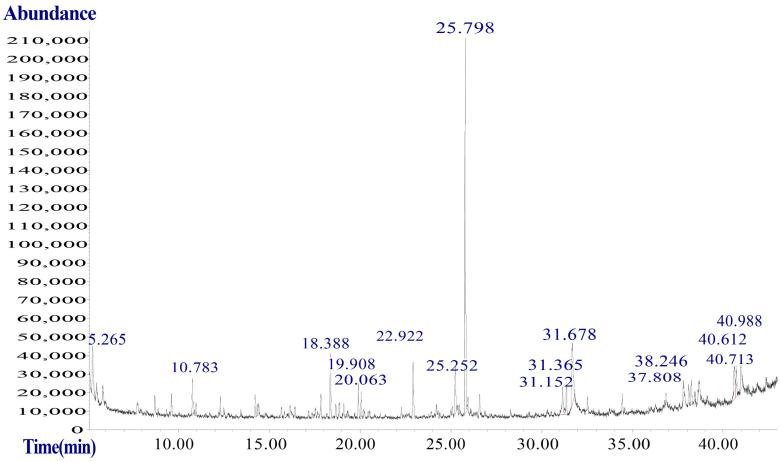
Total ion flow diagram of SLPE.

Figure 7 demonstrates the total ion flow diagram of SLPE analyzed by GC-MS. Table 1 demonstrates the chemical composition of SLPE analyzed by GC-MS. Compound 2,4-DTBP has the highest content. 2,4-DTBP is often a substantial ingredient in plants or essential oils, and its biological origins and actions have been extensively researched. It is mostly found in bacteria, fungi, diatoms, mosses, ferns, gymnosperms, dicots, monocots, and mammals. Biological actions include antioxidant, anti-inflammatory, antibacterial, antiviral, and antifungal properties, among others [26]. Sweet potato extract contains 2,4-DTBP, which protects against hydrogen peroxide-induced oxidative stress in a pheochromocytoma cell line (PC12) and animals. The administration of 2,4-DTBP enhanced the alternating behavior of mice injected with amyloid β-peptide [27].

**Figure 8:**
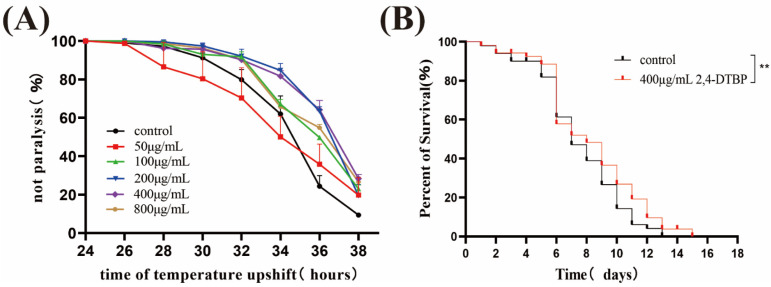
(**A**): Effects of 2,4-DTBP on the paralysis of *C. elegans* 4176. (**B**): Effects of 2,4-DTBP on the longevity of nematodes after heat shock. ** Significantly different from the control worms (*p* < 0.01).

Figures S3 and S4 show the hydrogen and carbon spectra of compounds 2,4-DTBP. As indicated in Figure 8, 400 μg/mL 2,4-DTBP significantly delayed the paralysis symptoms in CL4176. Heat shock is a series of high-temperature systemic acute inflammatory responses produced by exposure of animals to hot environments, which can lead to shock or even death of the animals [28]. The survival of N2 nematodes showed a decreasing trend with time at 37 °C. Still, the survival time of nematodes treated with compound 2,4-DTBP increased significantly when compared to the control, suggesting that the compound has a significant protective effect on nematodes in heat-stress environments.

## 3. Discussion

The production and deposition of Aβ is considered to be the main cause of the pathogenesis of AD, so reducing the production of Aβ and attenuating the toxicity of Aβ have become the main research direction for the treatment of AD [29]. Therefore, we used the CL4176s model study to screen drugs targeting Aβ and to investigate the protective effects and mechanisms of the natural product *S. leucantha* against Aβ-induced neurodegenerative diseases and found that 400 μg/mL of SLPE with compound 2,4-DTBP effectively delayed the symptoms of nematode paralysis and reduced Aβ-induced toxicity with the most significant effect. The treatment also prolonged the nematode paralysis symptoms. The treatment also prolonged the lifespan of nematodes without affecting motility and reproduction. Although the underlying molecular mechanisms need to be further investigated, this suggests that SLPE has the potential to be developed as a drug to alleviate the symptoms of AD and provides a theoretical foundation and experimental basis for the future treatment of AD. *C. elegans* has emerged as the most promising in vivo model for the study of AD, using bioinformatics methods to determine that 60–80% of nearly 20,000 genes are conserved in humans and that 12 of the 17 known signal transduction pathways are conserved in *C. elegans* [30]. *C. elegans*’ unequaled advantage as a model for human illness research and drug screening is due to the similarities between its regulatory systems and those of humans.

*C. elegans* has several phase I and II enzymes involved in xenobiotic metabolism in most organisms, such as P450-dependent monooxygenases, dehydrogenases, UDP-glucuronosyltransferases, and glutathione transferases [31]. Their expression is up-regulated in long-lived nematode larvae, and they are involved in the metabolism and elimination of potentially toxic endophytes and xenobiotics, which are major contributors to Aβ toxicity and molecular damage in nematode senescence [32]. In the biosynthesis of amino acids (BIA) pathway, amino acids play an important role in protein synthesis, glycoconversion, energy release, and induction of internal antioxidant responses by promoting glutathione synthesis [33]. Glutathione synthesis (GSH) in the glutathione metabolism pathway protects cells from oxidative stress by scavenging excess free radicals and thus plays a crucial role in antioxidant protection within nematodes [34]. Studies have shown that anti-AD activity is closely related to antioxidant activity [35]. Oxidative–antioxidant imbalance in the blood may be an early indicator of AD, and a balanced intake of antioxidants may prevent the development of AD [36]. Therefore, determining whether SLPE has an antioxidant effect has become the focus of the next research.

According to the idea of oxidative damage in senescence, a chain reaction of lipid peroxidation caused by the interaction of ROS (reactive oxygen species) with lipids exacerbates the original oxidative damage. The end products of lipid peroxidation, notably electrophilic aldehydes such as 4-HNE (4-hydroxy non-2-enal), are effectors that work in conjunction with ROS, leading to molecular damage and, eventually, senescence [37]. *Gst-5* has a role in the detoxification route of 4-HNE, a highly reactive neurotoxic byproduct of lipid peroxidation that has been linked to the development and progression of Alzheimer’s and Parkinson’s illnesses. It interacts with and changes several macromolecules, including proteins, DNA, RNA, and lipids, causing harmful consequences that are eventually involved in Alzheimer’s disease (AD) pathology [38]. Alzheimer’s patients had considerably higher amounts of free and protein-bound 4-HNE in the brain and cerebrospinal fluid than age-matched controls [39]. RNAi screens have demonstrated that *gst-5* is catalytically active in the binding of 4-HNE to glutathione. Knockdown of *gst-5* reduces the ability of nematode homogenates to bind 4-HNE to glutathione, leading to increased levels of 4-HNE–protein adducts, and loss of the gene product of *gst-5* also leads to a reduction in nematode lifespan [40]. The findings of this paper corroborate this result.

The heat shock response (HSR) is a physiological stress response triggered by misfolded cell membrane proteins that seek to restore protein folding or protein homeostasis. *C. elegans* has a unique and important role in HSR research because it can be quantified at the molecular, cellular, and organismal levels. Thus, changes at the molecular level may be observed at the cellular level, while their effects on physiology can be assessed at the organism level [41]. In *C. elegans*, HSR activation leads to overexpression of heat shock genes such as *hsp-70* and *hsp-16.2*. Many heat shock proteins (HSPs) operate as molecular chaperones that restore protein folding or protein homeostasis by directly interacting with misfolded or damaged proteins [42]. In the AD nematode model, *hsp-16.2* encodes HSP-16, which directly interacts with Aβ and alters its oligomerization pathway, thereby reducing toxicant formation [43]. Consistent with this, we found that SLPE up-regulated the expression level of *hsp-70* in CL4176 nematodes and that the compound 2,4-DTBP prolonged the lifespan of *C. elegans* under heat-stress conditions. This suggests that HSP plays an important role in the protective effect of SLPE. Techniques such as RNAi should be used subsequently to reveal further the involvement of HSP and other pathways in the effect of SLPE on Aβ-induced paralysis in the AD nematode model.

## 4. Materials and Methods

### 4.1. Plant Materials and Nematode Strains

The over-ground part of *S. leucantha* was harvested in July 2021 from Southwest Forestry University. After drying and crushing, the plant was refluxed with ethanol to obtain hot extract (SLR) and ultrasonically extracted at room temperature to obtain cold extract (SLL). The aerial part of *S. leucantha* 5 kg was dried, crushed, and refluxed with ethanol heated by water bath 3 times, each time for 3 h. The material–liquid ratio was 1:10, concentrated by rotary evaporator to obtain SLR 436.02 g. The SLR was dissolved in the water and then extracted with petroleum ether to obtain SLPE (65.48 g) with ethyl acetate to obtain SLEE (108.4 g), and the remainder was water extract (SLWE). The SLPE was separated by silica gel chromatography to obtain different flow fractions detected by thin layer chromatography (TLC), and the same components were combined to obtain SLPE-1, SLPE-2, SLPE-3, SLPE-4, SLPE-5, and SLPE-6. 2,4-Di-tert-butylphenol (97%) was from Shanghai Aladdin Biochemical Technology (Shanghai, China).

*E. coli* OP50 was used as food for nematodes and was nurtured on Nematode growth medium (NGM) solid medium at a constant temperature until the oviposition stage for synchronization to obtain L1-stage larvae for spare.

NGM solid medium 1000 mL: peptone 2.5 g; NaCl 3 g; agar 17 g/L; ddH_2_O was dissolved and fixed to 1000 mL and then dispensed in 500 mL conical flasks to add 300 mL of each of the above media. After autoclaving, filtered and sterilized 1 M phosphate buffer 7.5 mL, 1 M MgSO_4_ 300 µL, 1 M CaCl_2_ 300 µL, and cholesterol (5 mg/mL) 300 µL were added, the conical flask was shaken to make all the solutions mix well, and poured into 9 cm 6 cm plates, ensuring no air bubbles were produced when pouring the plate.

### 4.2. Bioactivity Assay for Alzheimer’s Disease-like Phenotype of Delaying Paralysis in C. elegans

The experimental model utilized in this study involved CL4176, which was genotyped as [*smg-1*(cc546)I;dvIs27X.]. A temperature-inducible system was incorporated into the worms, enabling monitoring of the *smg-1* mRNA during the experiment. The *smg-1* temperature-inducible system was activated when the worms were placed in a thermostat incubator at 16 °C. Under these conditions, the system effectively recognized and degraded the Aβ gene containing an incorrect 3′ non-coding region [44]. Following the initial incubation at 16 °C, the worms were transferred to an incubator maintained at 25 °C, causing the inactivation of the *smg-1* temperature-inducible system. At this elevated temperature, the mRNA for Aβ1-42 was expressed and accumulated in the myoblasts of CL4176. Consequently, toxic Aβ aggregates were formed, leading to a gradual loss of locomotor activity over time, ultimately resulting in paralysis [45].

Each medium was inoculated with 50–100 L1-stage CL4176 nematodes. They were placed in the incubator at 16 °C for normal growth and development and transferred to the incubator at 25 °C for continued cultivation for 24 h at constant temperature for 33 h before counting the paralyzed nematodes with a body microscope at intervals of 2 h, with three replicates in each group [46].

### 4.3. Phenotypic Experiments

Lifespan: At least 3 replicates per group. The NGM medium of the experimental group was supplemented with 400 μg/mL SLPE, and the control group had the same concentration of DMSO. The final concentration of DMSO did not exceed 0.1% after the addition of NGM [47]. Each NGM medium was inoculated with 50–100 synchronized L1-stage N2 larvae and incubated at 25 °C. The number of nematodes was counted every 24 h interval from the beginning of the incubation until all the nematodes died. The number of nematodes was counted at 24 h intervals from the beginning of the L1 stage culture until all nematodes died. When the nematodes grew to the point of adult spawning, the original adults were transferred to new culture plates to continue counting to remove the effect of nematode zygotes.

Fertility: The drug treatment was the same as the lifespan experiment. When the nematodes were cultured to the young adult stage, one nematode was randomly picked and placed in a new medium, and the number of nematode progeny was counted after continuing to culture for 48 h at 25 °C.

Body length: The drug treatment was the same as the longevity experiment, incubated at 25 °C for 24 h and 36 h, and the body length of 10 N2 nematodes were recorded in each plate under the microscope, as follows: some of the nematodes to be tested were rinsed in centrifuge tubes with M9 buffer, a drop of nematode liquid was placed on the slide and fire for 5 s, and then the lengths of the nematodes were measured under the microscope after they stiffened.

Pharyngeal pumping rate and motility: The drug treatment was the same as that of the lifespan experiment. After nematode inoculation, nematodes were cultured at 25 °C for 24 h and 36 h and randomly picked on slides with M9 buffer, and the pharyngeal pump activity and the number of head bobbing of the nematodes were measured under the microscope within 1 min.

### 4.4. PPI Network Construction

Protein–protein interaction (PPI) was used to examine the link between important genes (*p* < 0.05). Protein interactions were predicted using the Search Tool for the Retrieval of Interacting Genes (STRING) database (https://www.string-db.org/ accessed on 9 March 2024). The PPI network was visualized using Cytoscape software version 3.10.2.

### 4.5. Transcriptome Sequencing and RNAi

A total of 400 μg/mL SLPE was collected when the nematodes were transferred to the incubator at 25 °C for 33 h and stored in the refrigerator at −80 °C after liquid nitrogen quick-freezing. The experimental group and the control group, with a total of twelve samples in the two groups, were commissioned to complete the transcriptome sequencing by Shanghai Zhongke New Life (Shanghai, China).

Feeding of *C. elegans* with the disrupting strain was used to disrupt the target gene, and the target gene was disrupted and propagated for two generations, which could be used for subsequent paralysis experiments with the disrupting strain.

### 4.6. GC-MS

The analysis used gas chromatography-mass spectrometry (GC-MS) (Agilent 7890b-5977b, Santa Clara, CA, USA). The chromatographic column was an HP-5MS flexible quartz capillary column, 30 m × 250 μm, with a film thickness of 0.25 μm. The initial column temperature was 50 °C, held for 1 min, then increased to 250 °C at 5 °C per minute, held for 5 min. The inlet temperature was 250 °C, and the carrier gas was helium, with a pre-column pressure of 50 ka, a carrier gas flow rate of 1.20 mL/min, and a shunt ratio of 50:1. The ionization mode was EI, with an electron energy of 70 V, and the scanning range was 20–500 a.m.u.

### 4.7. Heat Shock Assays

Heat shock assays followed the methodology outlined by Li, with minor modifications [48]. The drug treatment administered to the nematodes was the same as that used in the lifespan experiment. Nematodes of the N2 strain were cultured at 25 °C until reaching the L4 stage. Subsequently, they were transferred to a culture condition of 37 °C, representing a heat-stress environment. The survival of the nematodes was monitored at one-hour intervals following the transfer until all nematodes eventually succumbed to the adverse conditions.

### 4.8. Statistical Analysis

The data were plotted using GraphPad Prism 9 software, the statistical method was a Student *t*-test, and the data were computationally collated using Excel 2019, including mean ± SEM and *p*-value. To compare the significance of the therapeutic effects of the extracts, *p* < 0.05 and *p* < 0.01 denote significant and highly significant differences, respectively.

## 5. Conclusions

Our study provides novel insights into the mechanisms underlying the delay of Aβ toxicity in Caenorhabditis elegans by *S. leucantha*, demonstrating its safety in preventing Alzheimer’s disease (AD). Experimental results revealed that *S. leucantha* extract (SLPE) effectively reduced Aβ deposition, prolonged the lifespan of the nematodes, and activated key pathway genes such as hsp, gst, and ins. Additionally, the active ingredient 2,4-DTBP exhibited the ability to delay nematode paralysis while also conferring resistance against heat shock. While *S. leucantha*’s activities have been widely investigated, our research is the first to uncover the protective effects of its active ingredient against Aβ toxicity and to systematically analyze its mechanistic action against AD. However, further verification through higher mammalian experiments is necessary to consolidate these findings.

## Figures and Tables

**Table 1:** Chemical constituents of SLPE.

Serial Number	Compounds	Matching Factor	Molecular Formula	CAS Number	Relative Content (%)
1	2,4-Di-tert-butylphenol	95.28641529	C_14_H_22_O	96-76-4	20.32
2	p-Xylene	92.08370814	C_8_H_10_	106-42-3	3.34
3	Mesitylene	91.02907647	C_9_H_12_	108-67-8	1.25
4	Cyclohexasiloxane, dodecamethyl-	90.74789501	C_12_H_36_O_6_Si_6_	540-97-6	1.29
5	Dodecane, 4,6-dimethyl-	90.6694043	C_14_H_30_	61141-72-8	3.19
6	Undecane, 5,7-dimethyl-	88.78228596	C_13_H_28_	17312-83-3	0.97
7	Nonane, 2-methyl-	88.39238821	C_10_H_22_	871-83-0	0.57
8	Dibenzylamine	88.06018078	C_14_H_15_N	103-49-1	11.45
9	Decane, 2,3,7-trimethyl-	87.51639754	C_13_H_28_	62238-13-5	1.17
10	2,6-Dimethyldecane	87.15292199	C_12_H_26_	13150-81-7	1.83

## Data Availability

All of the figures used to support the findings of this study are included within the article.

## Supplementary Materials

The supporting information can be downloaded at https://www.mdpi.com/article/10.3390/ijms251810188/s1.
