# Supplementary material for: Components in SLPE Alleviate AD Model Nematodes by Up-Regulating Gene gst-5
Source: Int J Mol Sci. 2024 Sep 23;25(18):10188. doi: 10.3390/ijms251810188 (PMC11432538; doi:10.3390/ijms251810188)

Figure S1 Volcano plot of genes altered by SLPE treatment in *C. elegans*.

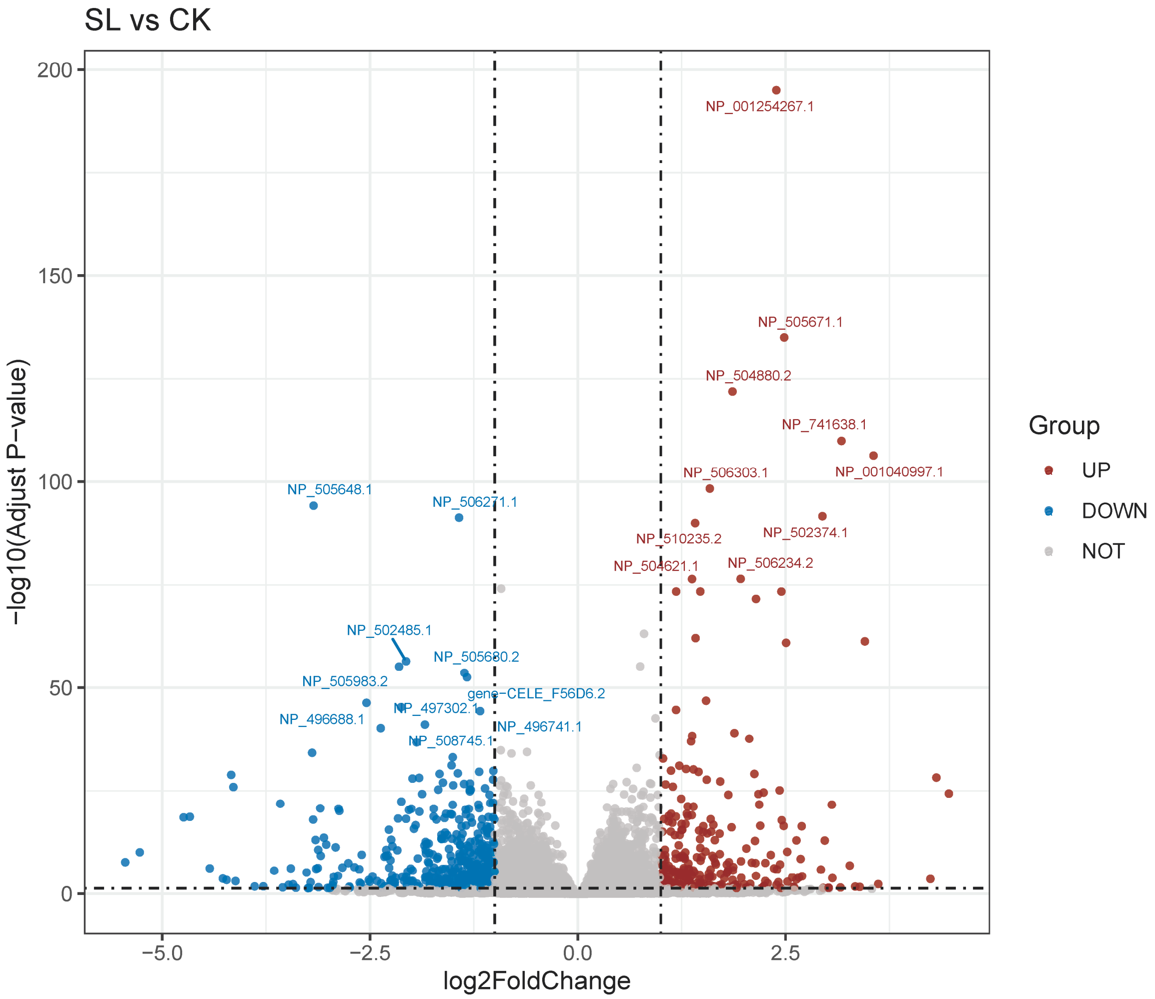

Figure S2 heatmap of genes altered by SLPE treatment in *C. elegans*.

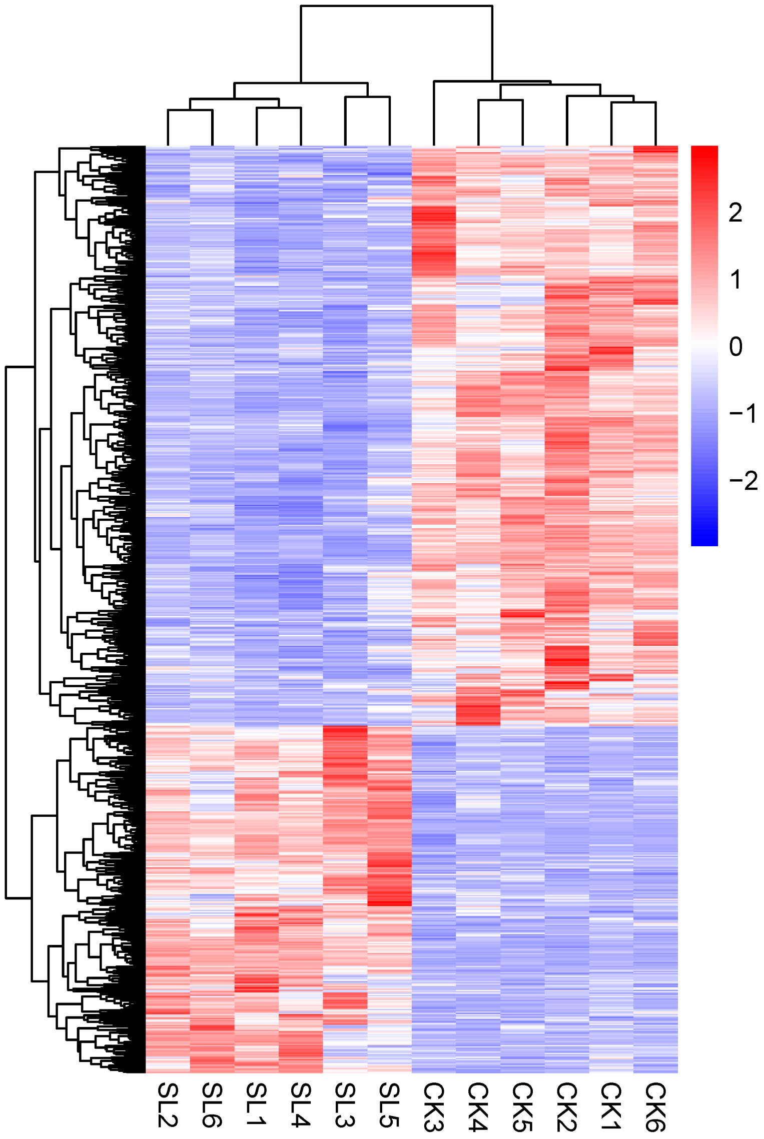

Figure S3 1H NMR (400 MHz, DMSO) 2,4-DTBP.

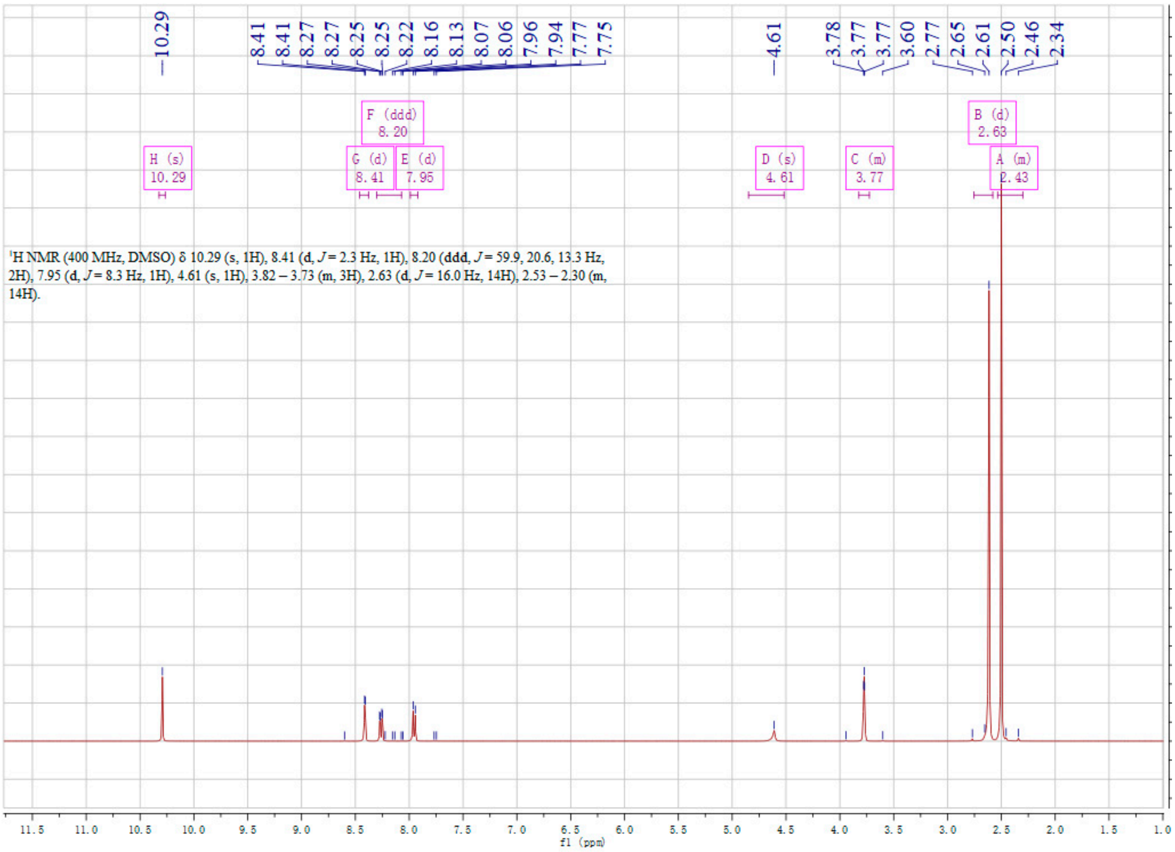

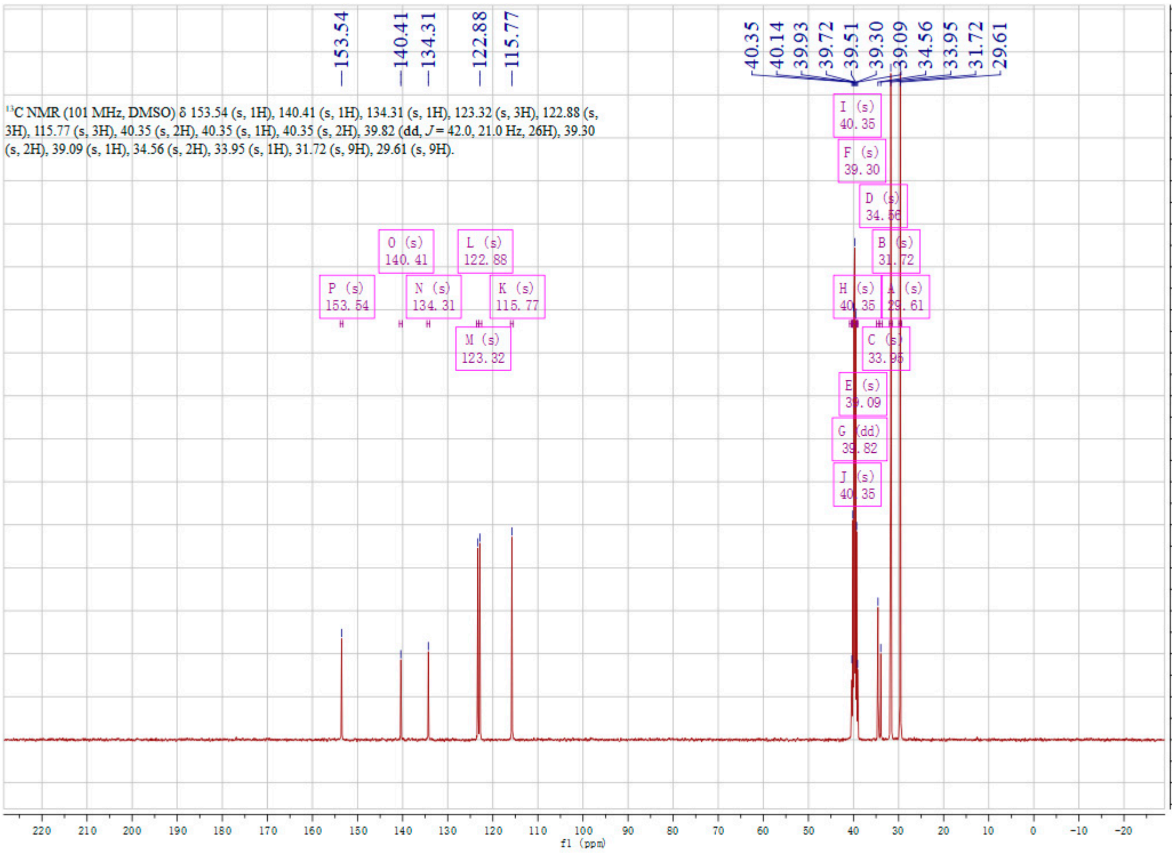

Figure S4 1C NMR (101MHz, DMSO) 2,4-DTBP.

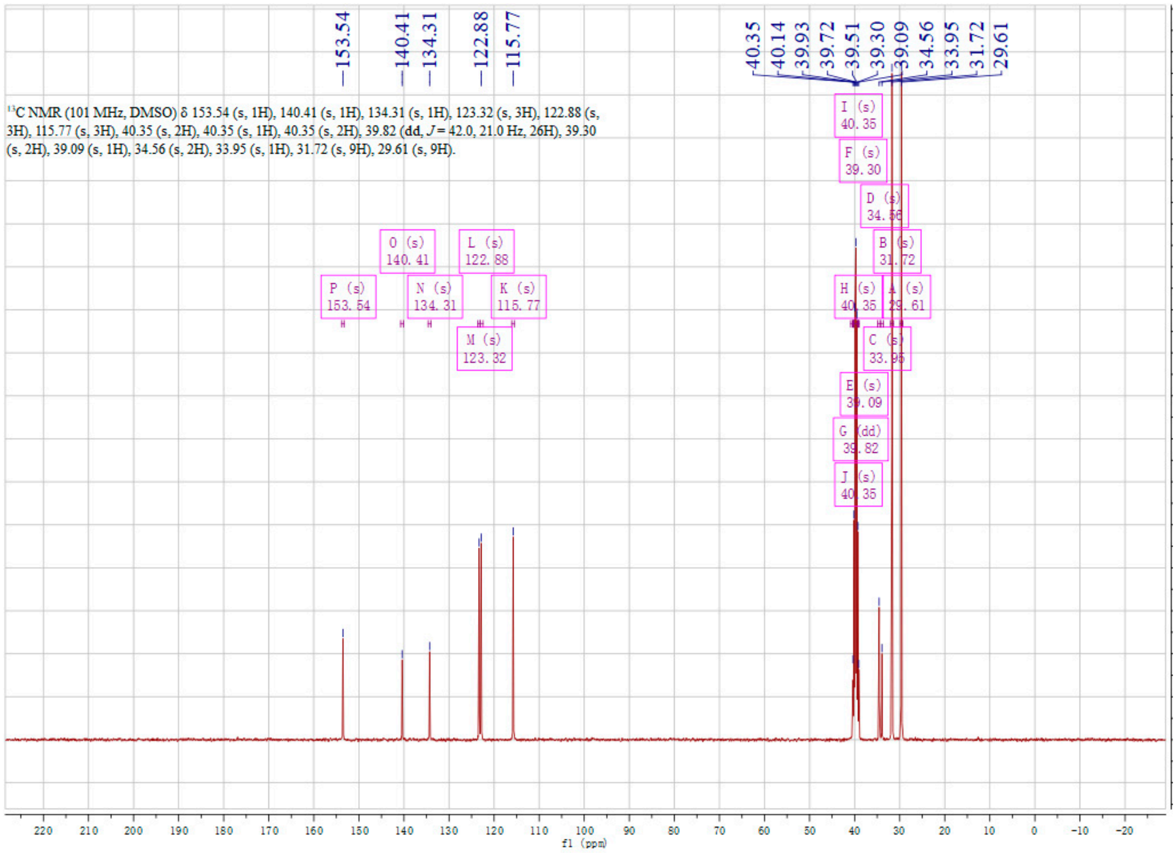

Supplement: Supplementary file 1 [file ijms-25-10188-s001.zip › ijms-3202777-Figures S1-S4.pdf]
